# A Pre-operative Nomogram for Prediction of Lymph Node Metastasis in Bladder Urothelial Carcinoma

**DOI:** 10.3389/fonc.2019.00488

**Published:** 2019-06-21

**Authors:** Xiaofan Lu, Yang Wang, Liyun Jiang, Jun Gao, Yue Zhu, Wenjun Hu, Jiashuo Wang, Xinjia Ruan, Zhengbao Xu, Xiaowei Meng, Bing Zhang, Fangrong Yan

**Affiliations:** ^1^Research Center of Biostatistics and Computational Pharmacy, China Pharmaceutical University, Nanjing, China; ^2^Department of Radiology, The Affiliated Nanjing Drum Tower Hospital of Nanjing University Medical School, Nanjing, China

**Keywords:** bladder cancer, lymph node metastasis, LNM signature, *MLL2* mutation, pre-operative nomogram

## Abstract

The status of lymph node (LN) metastases plays a decisive role in the selection of surgical procedures and post-operative treatment. Several histopathologic features, known as predictors of LN metastasis, are commonly available post-operatively. Medical imaging improved pre-operative diagnosis, but the results are not fully satisfactory due to substantial false positives. Thus, a reliable and robust method for pre-operative assessment of LN status is urgently required. We developed a prediction model in a training set from the TCGA-BLCA cohort including 196 bladder urothelial carcinoma samples with confirmed LN metastasis status. Least absolute shrinkage and selection operator (LASSO) regression was harnessed for dimension reduction, feature selection, and LNM signature building. Multivariable logistic regression was used to develop the prognostic model, incorporating the LNM signature, and a genomic mutation of *MLL2*, and was presented with a LNM nomogram. The performance of the nomogram was assessed with respect to its calibration, discrimination, and clinical usefulness. Internal validation was evaluated by the testing set from the TCGA cohort and independent validation was assessed by two independent cohorts. The LNM signature, which consisted of 48 selected features, was significantly associated with LN status (*p* < 0.005 for both the training and testing sets of the TCGA cohort). Predictors contained in the individualized prediction nomogram included the LNM signature and *MLL2* mutation status. The model demonstrated good discrimination, with an area under the curve (AUC) of 98.7% (85.3% for testing set) and good calibration with *p* = 0.973 (0.485 for testing set) in the Hosmer-Lemeshow goodness of fit test. Decision curve analysis demonstrated that the LNM nomogram was clinically useful. This study presents a pre-operative nomogram incorporating a LNM signature and a genomic mutation, which can be conveniently utilized to facilitate pre-operative individualized prediction of LN metastasis in patients with bladder urothelial carcinoma.

## Introduction

Bladder cancer is the 9th most prevalent cause of cancer worldwide and the 2nd most common genitourinary malignancy, with transitional cell carcinomas comprising about 90% of primary bladder tumors (1). In 2019, ~80,470 new cases and 17,670 deaths of bladder cancer were estimated to occur in the United States (2). Previous research has revealed that Lymph node (LN) involvement—which is frequently found in bladder cancer—possesses prognostic implications, and both the pathological stage of primary bladder tumor and the presence of LN metastasis are considered the most important determinants of survival in bladder cancer patients undergoing radical cystectomy (3). Early and accurate identification of LN metastasis holds significance in improving patient triage and management (4) and may suggest potential alteration of the lymphadenectomy template in patients who undergo surgery. In cases where local staging is equivocal, it also expedites care for patients, particularly when nodal disease can be definitively identified (5–7). Pre-operative knowledge of LN metastasis provides valuable information about the necessity of adjuvant therapy and the adequacy of surgical resection, thereby aiding pretreatment decision-making, but unfortunately, most histopathologic findings identified as predictors of LN metastasis cannot be observed pre-operatively. That is to say, the status of LN metastases plays a decisive role in the selection of surgical procedures and post-operative treatment. Reliable and robust methods for pre-operative assessment of LN status (8) have been continuously explored. Fine-needle aspiration lymphangiography has been evaluated in several investigations but failed to show reliability due to a high false negative rate (9). Only a few studies have appraised positron emission tomography (PET) and its ability to detect LN metastases in bladder cancer, but the conclusions have been largely disappointing (10). Additionally, computed tomography (CT) revealed a high false negative rate of 21% (11). Next-generation sequencing technology has brought massively high throughput sequencing data to bear on research questions with low cost, which enables us to decipher the difference of bladder cancer in terms of status of LN metastasis in a genomic level. Currently, the analysis strategy for multiple biomarkers has evolved from individual analyses to combined analysis of a panel of biomarkers that constitute a signature, which appears to be a most promising approach and powerful enough to innovate clinical management (12, 13). Therefore, this study aims to develop and validate a pre-operative nomogram that incorporates a LN-metastasis signature and genomic mutations for individualized pre-operative prediction of LN metastasis in patients with bladder cancer.

## Materials and Methods

### Patients and Samples

Molecular data were obtained from The Cancer Genome Atlas Project (TCGA) patients diagnosed with bladder urothelial carcinoma. Transcriptome HTSeq-counts data of TCGA-BLCA project was obtained from the Genomic Data Commons (https://portal.gdc.cancr.gov/) using the R package “*TCGAbiolinks”* (14). Somatic mutation profiling and detailed clinicopathological information were downloaded from cBioPortal (http://www.cbioportal.org/datasets). For the purpose of the present study, 196 samples were selected as the TCGA cohort including 49 samples with LN metastasis only (LN+) and 147 without any metastasis (LN–). Two independent cohorts were gathered for validation including one obtained from Gene Expression Omnibus (https://www/ncbi.nlm.nih.gov/geo/) (GEO cohort) by using R package “*GEOquery”* with a query of GSE106534 and another that is publicly available through the Memorial Sloan-Kettering Cancer Center (MSKCC cohort) cBioPortal for Cancer Genomics. The GEO cohort contains five LN+ and five LN– bladder tissues, of which RNA was extracted and hybridized on an Illumina Hiseq 2500 (13). The MSKCC cohort contains 58 tumor samples with Agilent microarray mRNA expression profiling (15). Survival information of the MSKCC cohort was obtained from the cBioPortal.

### Data Preprocessing for Transcriptome HTSeq-Counts

Ensembl ID for genes (protein-coding mRNAs) was annotated in GENCODE27 to generate Gene Symbol name. Gene type of protein-coding was selected for mRNAs. We calculated the number of fragments per kilobase of non-overlapped exons per million fragments mapped (FPKM) first and subsequently transferred FPKM into transcripts per kilobase million (TPM) values, which are more similar to those resulting from microarrays and more comparable between samples (16). To reduce noise, only mRNAs with TPM value equal to or above one in at least 10% of the samples were kept for downstream analysis.

### Differential Expression and Functional Enrichment Analysis

Differential expression analysis was performed by R package “*DESeq2”* with the standard comparison mode between the two experimental conditions (17). *P*-values were adjusted for multiple testing with an embedded Benjamini-Hochberg procedure in the package. Gene set enrichment analysis (GSEA) was performed by R package “*clusterProfiler*” (18, 19) to impute functional pathway enrichment for the LN+ and LN– groups by mRNA expression profile.

### Genetic Analysis on Somatic Mutation

We used MutSigCV_v1.41 (20) (www.broadinstitute.org) to infer significant cancer mutated genes (*q* < 0.05) across the two classes currently identified with default parameters. Tumor mutation burden was computed by summing all kinds of non-silent mutation. Mutation landscape oncoprint was drawn by R package “*ComplexHeatmap”* (21). Significant frequent non-silent mutations were identified by independent test between the LN+ and LN– groups with a *p* < 0.05.

### Feature Selection

The TCGA cohort of 196 samples was randomized into two sets based on 10-fold stratified sampling, where the training set included 9 folds of LN+ samples and LN− samples and the testing set included the rest, 1 fold with 5 LN+ samples and 15 LN− samples. Least absolute shrinkage and selection operator (LASSO) regression, which is often applied as a dimensionally reduction technique, was performed on the training set to select primary predicative features. Ten-fold cross validation was performed to tune the optimal value of lambda (λ) that gives the minimum mean cross-validated error. A score was calculated for each sample via a linear combination of the selected features, namely LNM signature, weighted by the corresponding coefficients. The potential association of the LNM signature with LN status was first assessed in the training set and then validated in the testing set by using the Mann-Whitney *U*-test.

### Development of an Individualized Prediction Model

Multivariable logistic regression analysis on the training set began with the following candidate predictors: LNM signature and significant frequent mutations. Those with respective *p* < 0.05 were retained in the prognostic model. A LNM nomogram was built by R package “*regplot”* as a quantitative tool for clinicians for individualized prediction of LN metastasis probability.

### Validation of the LNM Nomogram and LNM Signature

Internal validation was performed using the testing set of the TCGA cohort with 20 samples. The logistic regression formula formed in the training set was applied to all samples in the testing set, with total points for each sample calculated. Logistic regression in the testing cohort was then performed by using the total points as a factor. Finally, the receiver operating characteristic curve (ROC) with area under the curve (AUC) and calibration curve were derived based on the regression analysis by using R packages “*pROC”* and “*rms”*. Independent validation for LNM-score was tested in the GEO and MSKCC cohorts. Since the mutation data were absent and several genes of the LNM signature failed to be mapped in independent cohorts, we harnessed unsupervised clustering to determine if LNM signature could help distinguish LN metastasis status in the GEO cohort and whether it was associated with overall survival (OS) or progression-free survival (PFS) in the MKSCC cohort. Supervised hierarchical clustering based on mapped LNM signature was performed by using R function hclust() via the Ward.D clustering method 1-Pearson's correlation distance, with *k* = 2 as the number of clusters. Expression profiling of mRNAs was transformed by log_2_(x+1) and median-centered before clustering.

### Statistical Analysis

All statistical tests were executed by R/3.5.2, with a χ^2^ or Fisher's exact test for categorical data when appropriate, a two-sample student's *t*-Test or Mann-Whitney *U*-test for continuous data when appropriate, a log-rank test Kaplan-Meier curve (22) and Cox regression (23) for survival analysis performed by R package “*survival*.” Survival of patients belonging to different defined groups was compared by the Kaplan-Meier Method, with *p*-value determined by the log-rank (Mantel-Cox) test. Fisher's exact test of independence was used to statistically test the association between categorical clinical information and LN metastasis status. For all statistical analysis, a two-sided *p* < 0.05 was considered statistically significant. Decision curve analysis was conducted to determine the clinical usefulness of the LNM nomogram by quantifying the net benefits at different threshold probabilities by using R package “*rmda”* (24, 25).

## Results

### Demographic Characteristics

**Figure 1:**
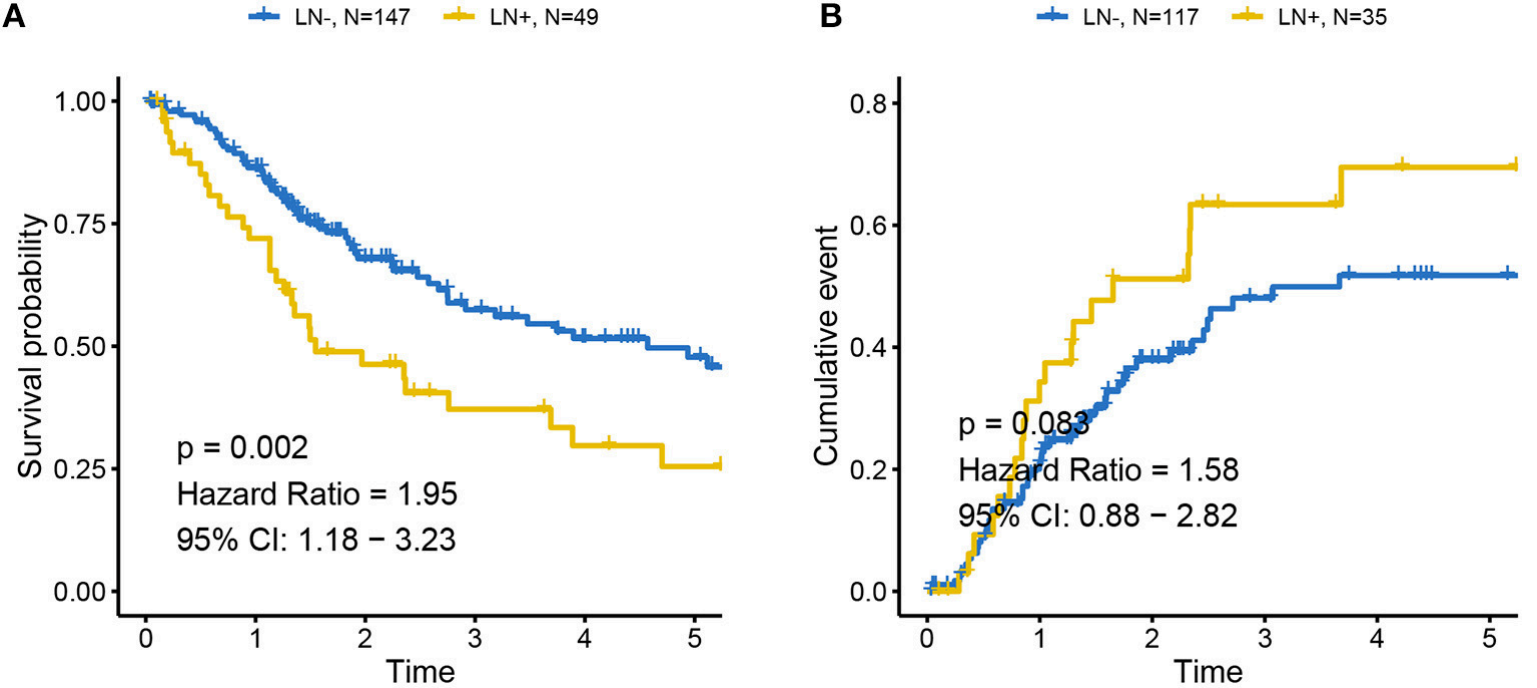
Association between LN metastasis status and patients' outcomes in TCGA cohort **(A)** for overall survival and **(B)** for progression-free survival. Tumors with LN+ demonstrated poor prognosis compared to LN– and a tendency could be observed where LN+ tumors presented higher recurrence rates. LN– was regarded as the reference for the calculation of HR.

The distributions of gender, age (dichotomized by median age of 69), BMI (trichotomized by WHO body mass index cut-off), pack-year of smoking history (dichotomized by median pack of 29), papillary type and histological grade were not different between LN+ and LN– samples. Race (*p* = 0.031), LN category (*p* = 1.30–13), metastasis category (*p* = 0.035), pathological stage (*p* = 7.04–11), lymph node examined number (dichotomized by median number of 27, *p* = 0.039), and tumor status (*p* = 0.0004) were significantly associated with LN metastasis status (Table 1). As expected, tumors with LN+ demonstrated poorer prognosis than LN– (*p* = 0.002, HR = 1.95, 95% CI = [1.18–3.23], Figure 1A) and a tendency could be observed where LN+ tumors presented a higher recurrence rate (*p* = 0.083, HR = 1.58, 95% CI = [0.88–2.82], Figure 1B).

**Table 1:** Demographic and clinicopathological characteristics of patients with bladder urothelial carcinoma (TCGA cohort, *n* = 196) based on LN metastasis status.

**Clinicopathogical parameters**	**Frequency (%)**	**TCGA-BLCA**		***P*-value**
		**LN+ (*n* = 49)**	**LN– (*n* = 147)**	
**Gender**				0.3615
Female	54 (28)	16	38	
Male	142 (72)	33	109	
**Age (years)**				1.0000
>69	92 (47)	23	69	
≤ 69	104 (53)	26	78	
**BMI**				0.4678
<18.5	6 (3)	0	6	
18.5–24	46 (23)	11	35	
>24	133 (68)	35	98	
Missing	11 (6)	3	8	
**Pack-year of smoking**				0.6761
>29	59 (30)	17	42	
≤ 29	59 (30)	14	45	
Missing	78 (40)	18	60	
**Race**				0.0107^*^
Not white	30 (15)	2	28	
White	163 (83)	47	116	
Missing	3 (2)	0	3	
**Histologic subtype**				0.2858
Non-Papillary	132 (67)	37	95	
Papillary	61 (31)	12	49	
Missing	3 (2)	0	3	
**Histologic grade**				0.1948
High	187 (95)	49	138	
Low	7 (4)	0	7	
Missing	2 (1)	0	2	
**Tumor stage**				0.0005^*^
T1 + T2	59 (30)	6	53	
T3 + T4	122 (62)	42	80	
Missing	15 (8)	1	14	
**Lymph node stage**				1.30e–13^*^
N0 + N1	140 (71)	17	123	
N2 + N3	45 (23)	32	13	
Missing	11 (6)	0	11	
**Metastasis stage**				0.0350^*^
M0	108 (55)	89	19	
M1	2 (1)	0	2	
Missing	86 (44)	58	28	
**Pathologic tumor stage**				7.04e–11^*^
I + II	65 (33)	0	65	
III + IV	130 (66)	49	81	
Missing	1 (1)	0	1	
**Examined lymph nodes**				0.0393^*^
>27	77 (39)	29	48	
≤ 27	85 (43)	19	66	
**Tumor status**				0.0004^*^
Tumor free	121 (62)	19	102	
With tumor	61 (31)	25	36	
Missing	14 (7)	5	9	

* *Fisher's exact test p < 0.05*.

### Overview of Differential Expression Results From LN+ and LN– Tumors

**Figure 2:**
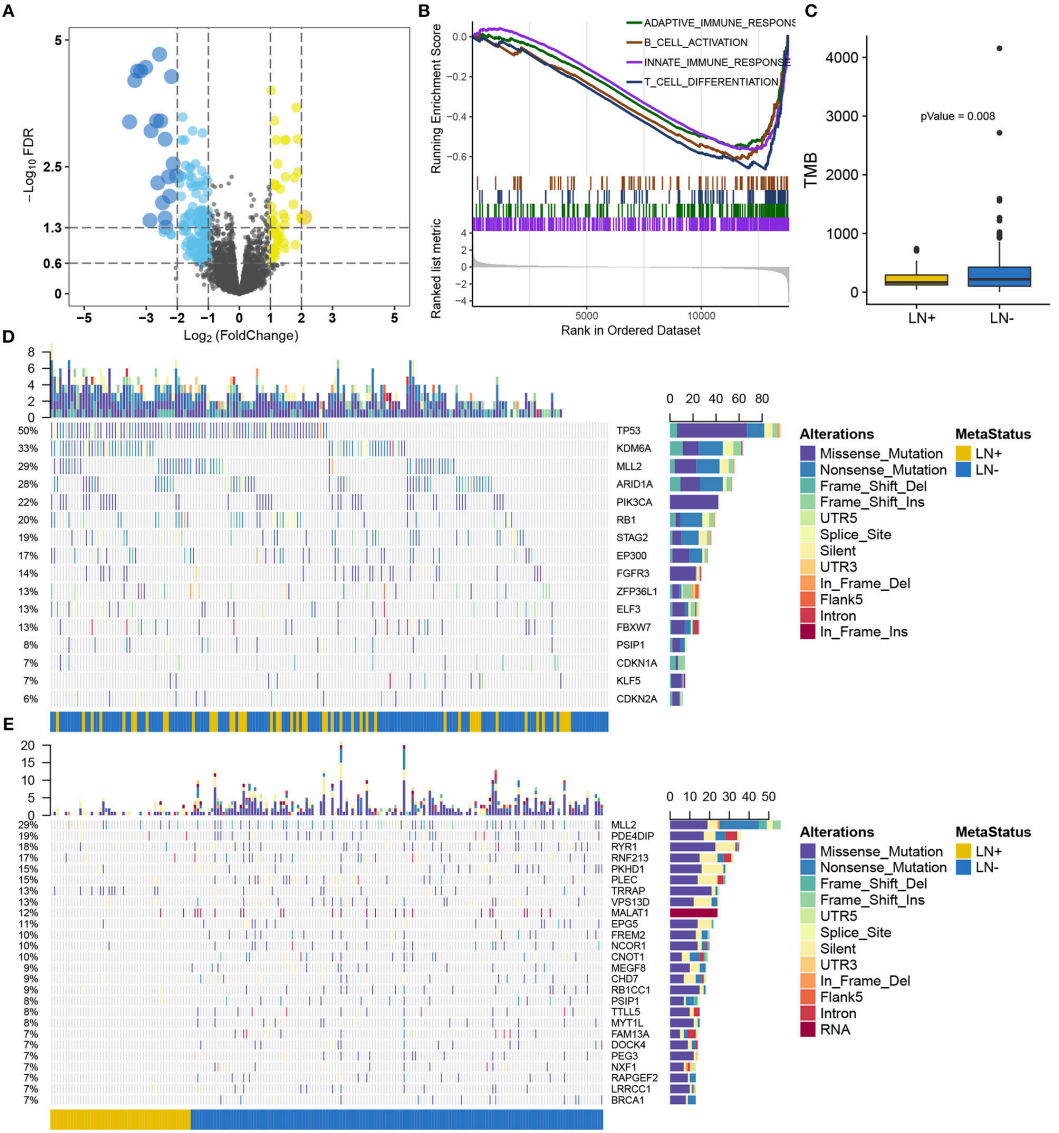
Overview of the molecular differences between LN+ and LN− tumors in the TCGA cohort. **(A)** Volcano plot for differentially expressed genes. **(B)** GSEA demonstrated down-regulated immune-related pathways in LN+ tumors. **(C)** Boxplot showed significantly lower TMB in LN+ tumors as compared to LN- tumors. Oncoprint for SMGs identified by MutSigCV shown in **(D)** and **(E)** depicted significantly differentially mutated genes based on LN metastasis status.

Supervised differential expression analysis using LN metastasis status as the group variable identified 180 differentially expressed genes (*p* < 0.05, false discovery rate (FDR) <0.05, Figure 2A; Supplementary Table S1). GSEA manifested a universal down-regulation of immune-related pathways in LN+ tumors as compared to LN– tumors (Figure 2B; Supplementary Table S2).

### Somatic Mutation Landscape Between LN+ and LN– Tumors

After filtering out non-silent mutation, tumors with LN+ exhibited a significant lower burden of mutation load as compared to LN– tumors (*p* = 0.008) (Figure 2C). MutSigCV identified 16 significantly mutated genes (SMGs, *q* < 0.05) for the present 193 samples with available mutation data (Supplementary Table S3), all of which were reported from previous research (26) (Figure 2D). We identified 716 genes with a mutation rate >5%, among which 26 genes were found to be differentially mutated between LN+ and LN– tumors by independent test (Figure 2E; Supplementary Table S4). By intersecting with SMGs, mutation of *MLL2* (also known as *KMT2D*) and PSIP1 were identified for constructing a predictive model.

### Feature Selection and LNM Signature Building

**Figure 3:**
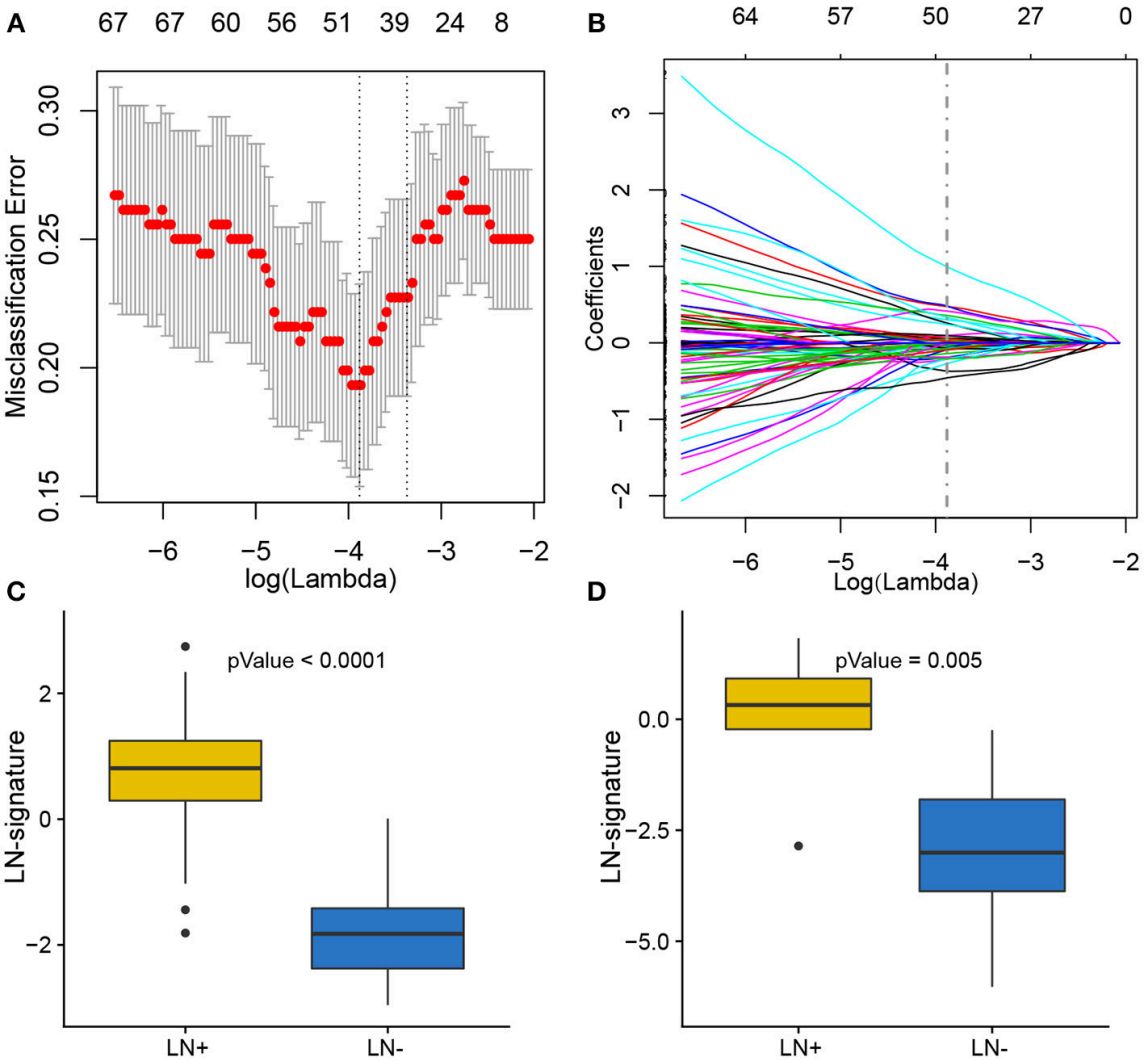
Feature selection using LASSO binary logistic regression model. **(A)** Tuning parameter λ (lambda) selection in the LASSO model used 10-fold cross-validation by minimum criteria. The misclassification error was plotted vs. log(λ). Dotted vertical lines were drawn at the optimal values by using the minimum criteria. A λ of 0.021 with log(λ) of −3.881 was chosen according to 10-fold cross-validation. **(B)** LASSO coefficient profiles of the 180 genes. A coefficient profile plot was produced against the log(λ) sequence. A vertical line was drawn at the value selecting by 10-fold cross-validation, where the optimal λ resulted in 48 non-zero coefficients. Distribution of calculated LNM signature vs. LN metastasis status for both training set and testing set were plotted by boxplot in **(C)** and **(D)**, respectively.

Of 180 differentially expressed genes, 48 features were selected on the basis of the training set of the TCGA cohort (see Materials and methods for more details), including 22 up-regulated genes and 26 down-regulated genes, as they were features with non-zero coefficients in the LASSO logistic regression model (Figures 3A,B; Table 2). These features are presented in LNM signature calculation formula (Supplementary Table S5). LNM signature was significantly higher in LN+ tumors as compared to LN– tumors both in the training (*p* < 2.2–16) and testing sets (*p* = 0.005) of the TCGA cohort (Figures 3C,D) and appeared to be an independent predictor for OS (*p* = 0.0264, HR = 1.13, 95% CI = [1.01–1.27]) by multivariate Cox regression integrating LNM signature, gender, age and histological grade of the entire TCGA cohort.

**Table 2:** Details of LASSO-selected genes in differential expression analysis.

**Gene symbol**	**Description**	**log2FC**	***P*-value**	**FDR**
SFTPA2	Surfactant protein A2	3.827	< 0.0001	< 0.0001
CES1	Carboxylesterase 1	2.571	< 0.0001	< 0.0001
NOS2	Nitric oxide synthase 2	−1.889	< 0.0001	< 0.0001
SCGB3A1	Secretoglobin family 3A member 1	1.835	< 0.0001	0.0002
MMP1	Matrix metallopeptidase 1	−1.824	< 0.0001	0.0003
AKR1B1	Aldo-keto reductase family 1 member B	1.123	< 0.0001	0.0004
VNN2	Vanin 2	−1.509	< 0.0001	0.0006
SPRR2F	Small proline rich protein 2F	−2.846	< 0.0001	0.0006
SLC39A2	Solute carrier family 39 member 2	1.881	< 0.0001	0.0009
UGT2B15	UDP glucuronosyltransferase family 2 member B15	−2.392	< 0.0001	0.0009
SUSD2	Sushi domain containing 2	1.187	< 0.0001	0.0009
ANPEP	Alanyl aminopeptidase, membrane	1.500	< 0.0001	0.0009
GALR2	Galanin receptor 2	1.468	< 0.0001	0.0009
GPAT2	Glycerol-3-phosphate acyltransferase 2, mitochondrial	−1.577	< 0.0001	0.0032
ID4	Inhibitor of DNA binding 4, HLH protein	−1.208	< 0.0001	0.0038
SLC10A4	Solute carrier family 10 member 4	−1.538	< 0.0001	0.0050
KISS1	KiSS-1 metastasis suppressor	1.486	< 0.0001	0.0050
LARP7	La ribonucleoprotein domain family member 7	−0.250	< 0.0001	0.0050
ALDH3A1	Aldehyde dehydrogenase 3 family member A1	1.775	< 0.0001	0.0051
ERVMER34-1	Endogenous retrovirus group MER34 member 1, envelope	−1.037	< 0.0001	0.0058
HBB	Hemoglobin subunit beta	−1.313	< 0.0001	0.0064
OSGIN1	Oxidative stress induced growth inhibitor 1	1.112	< 0.0001	0.0067
GUF1	GUF1 homolog, GTPase	−0.344	< 0.0001	0.0067
EFCAB1	EF-hand calcium binding domain 1	−1.409	< 0.0001	0.0075
METTL7B	Methyltransferase like 7B	1.319	< 0.0001	0.0087
ATP6V0A1	ATPase H+ transporting V0 subunit a1	0.370	0.0001	0.0111
AP002990.1	UNCHARACTERIZED Protein^*^	0.463	0.0001	0.014
KLHDC9	Kelch domain containing 9	0.917	0.0001	0.0141
GAS6	Growth arrest specific 6	0.965	0.0001	0.0164
HS3ST1	21eparin sulfate-glucosamine 3-sulfotransferase 1	−0.818	0.0001	0.0173
FAM219A	Family with sequence similarity 219 member A	0.396	0.0001	0.0185
CD177	CD177 molecule	−1.352	0.0002	0.0256
KLRF1	Killer cell lectin like receptor F1	−1.008	0.0002	0.0274
FOXL2	Forkhead box L2	1.413	0.0002	0.0274
FMNL2	Formin like 2	−0.700	0.0002	0.0278
TGS1	Trimethylguanosine synthase 1	−0.344	0.0002	0.0278
SKIL	SKI like proto-oncogene	0.546	0.0002	0.0284
RPRM	Reprimo, TP53 dependent G2 arrest mediator homolog	−1.179	0.0002	0.0288
MED28	Mediator complex subunit 28	−0.253	0.0003	0.0312
KRT23	Keratin 23	1.461	0.0003	0.0318
EDEM1	ER degradation enhancing alpha-mannosidase like protein 1	0.417	0.0003	0.0322
GRAMD2A	GRAM domain containing 2A	−1.054	0.0004	0.0366
SMOC1	SPARC related modular calcium binding 1	−1.346	0.0005	0.0408
KPNA7	Karyopherin subunit alpha 7	1.022	0.0005	0.0421
NOP14	NOP14 nucleolar protein	−0.271	0.0005	0.0431
ATG4C	Autophagy related 4C cysteine peptidase	−0.252	0.0006	0.0471
CRH	Corticotropin releasing hormone	−2.369	0.0006	0.0491
C2orf88	Chromosome 2 open reading frame 88	−0.805	0.0006	0.0492

* *Novel transcript annotated by GeneCards*.

### Supervised Clustering by Using LNM Signature in Two Independent Cohorts

**Figure 4:**
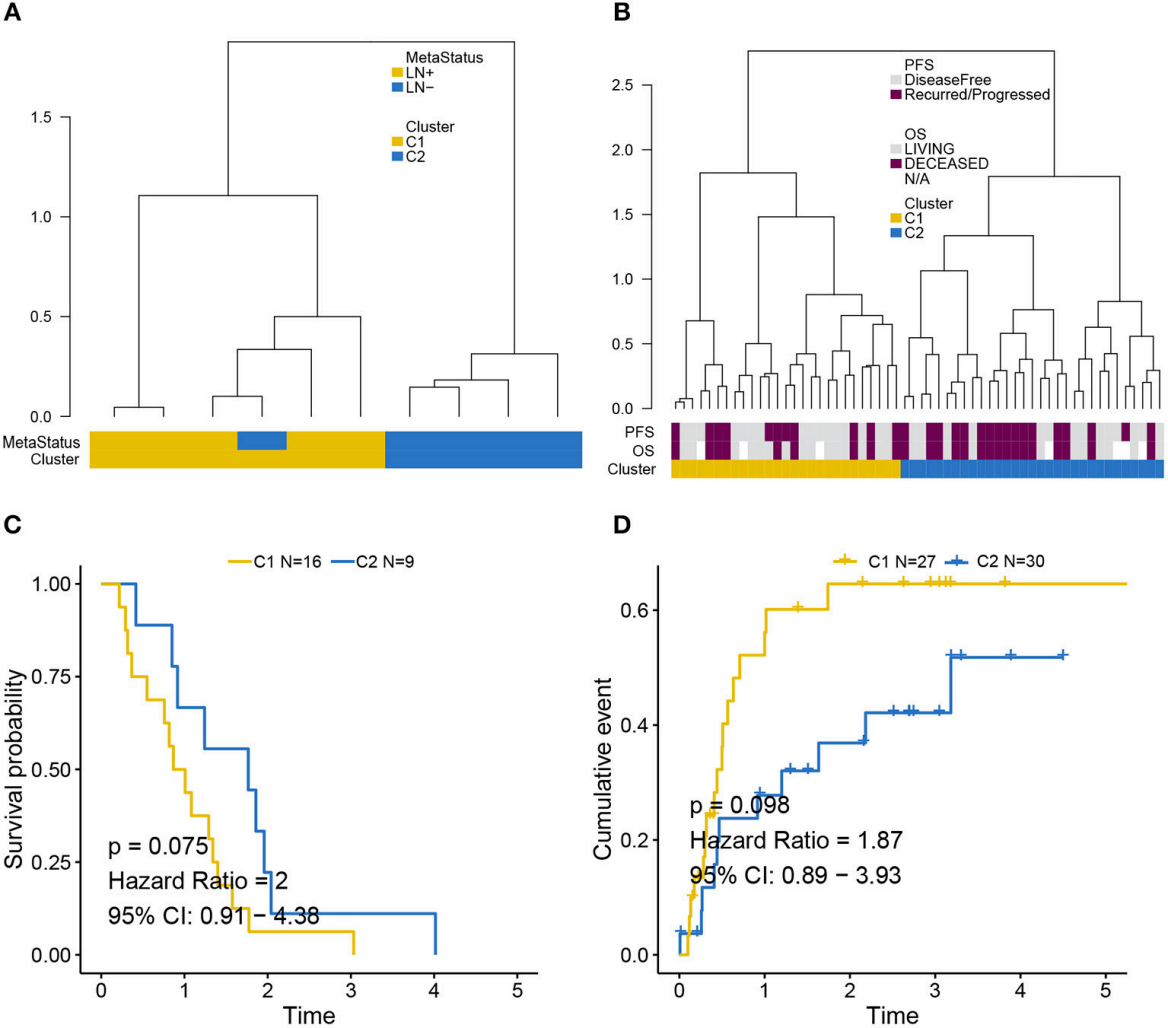
Validation of LNM signature via supervised clustering. **(A)** Dendrogram created by supervised hierarchical clustering using the GEO cohort significantly distinguished LN metastasis status (*p* = 0.048) and a dendrogram created for the MSKCC cohort in **(B)** identified two clusters with a tendency whereby LNM signature was associated with **(C)** OS (*p* = 0.075) and **(D)** PFS (*p* = 0.098). Cluster C2 was regarded as reference when calculating HR.

Independent validation for LNM signature was performed in the GEO (Figure 4A) and MSKCC cohorts (Figure 4B) by supervised hierarchical clustering where samples in the GEO cohort could be distinguished according to LN metastasis status (*p* = 0.048), and a tendency could be observed that LNM signature was associated with OS (*p* = 0.075) (Figure 4C) and PFS (*p* = 0.098) (Figure 4D) in the MSKCC cohort.

### Development of an Individualized Prognostic Model

**Figure 5:**
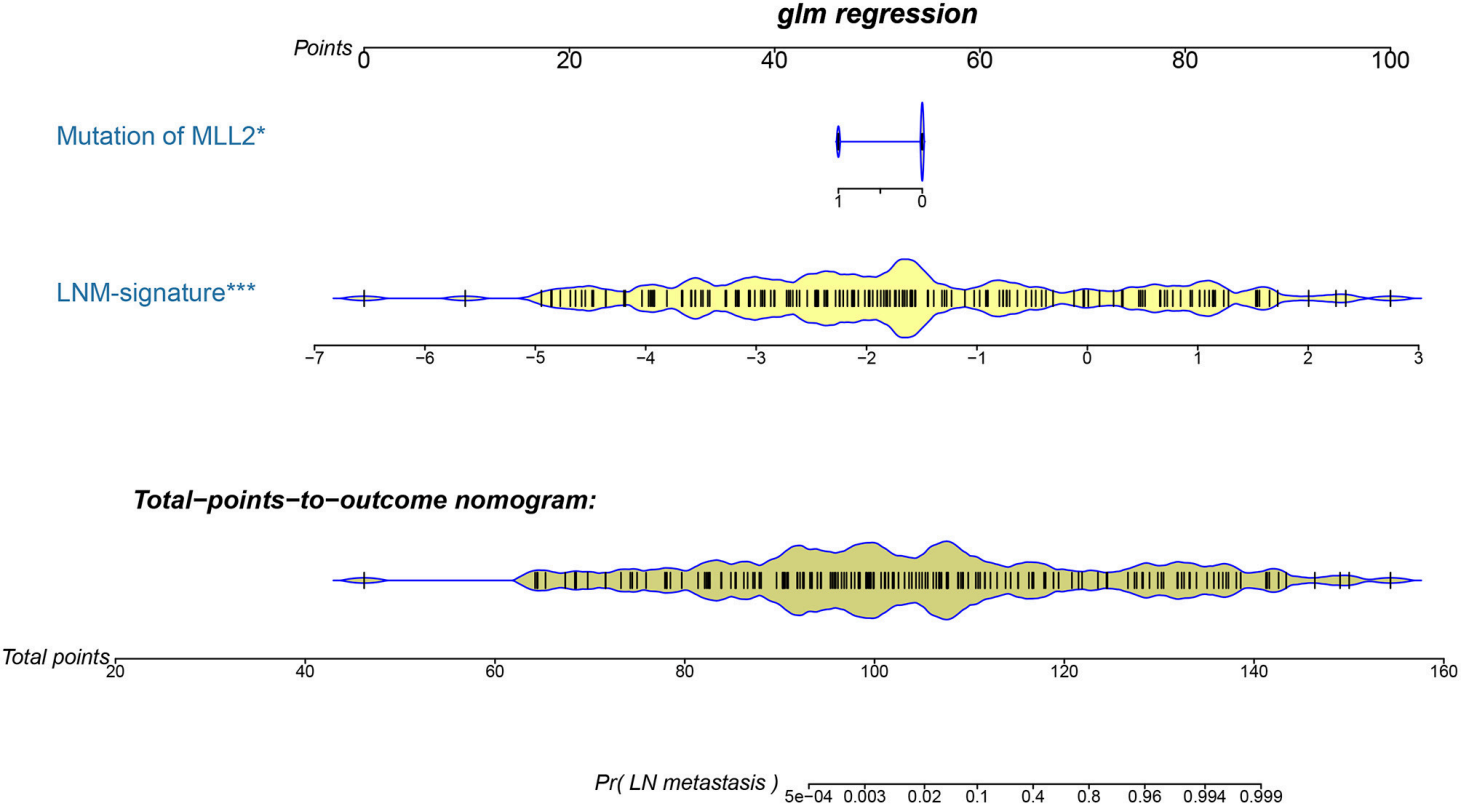
The developed pre-operative nomogram. The LNM nomogram was built in the training set of the TCGA cohort, with the LNM signature and genomic mutation of MLL2 incorporated.

Logistic regression analysis identified the pre-operative features of LNM signature and *MLL2* mutation as independent predictors (Table 3). We also considered other pre-operative clinical variables when designing the prognostic model, and interestingly, only LNM signature and *MLL2* mutation survived in the full model with *p* < 0.05 in the logistic regression (Supplementary Table S6). Thus, we further removed these variables in this study not only due to the insignificance of other variables, but also because we hope that patients could accept the acquisition of these measurements relatively easily. For this reason, we considered that in most cases, pathologic stage and detailed TNM classification should be detected by biopsy, an invasive procedure that may be much less acceptable than pre-designed multi-gene assay that may only need a small amount of blood. Hence, a model incorporating these two features was developed and presented as a LNM nomogram (Figure 5). Predictions made by calibration curve of the nomogram for LN metastasis conformed well to observations in the training set, with a Hosmer-Lemeshow test suggesting no departure from perfect fit (*p* = 0.973).

**Table 3:** Summary of logistic regression model integrating LNM signature and genomic mutations.

**Intercept and variables**	**Baseline model**			**Final model**		
	**β**	**Odds ratio (95% CI)**	***P-*value**	**β**	**Odds ratio (95% CI)**	***P*-value**
(Intercept)	2.0623	–	0.004	2.0690	–	0.004
LNM signature	3.2557	18.29 (6.16–54.28)	<0.001	3.2753	18.29 (6.16–54.28)	<0.001
MLL2 mutation	−2.4800	0.32 (0.12, 0.8)	0.03	−2.4870	0.32 (0.12, 0.8)	0.030
PSIP1 mutation	−13.3239	0 (0, Inf)	0.99	NA	NA	NA

*β is the estimate coefficient and NA means the corresponding variable was removed from the model fitting*.

### Validation of the LNM Nomogram and Its Clinical Usefulness

**Figure 6:**
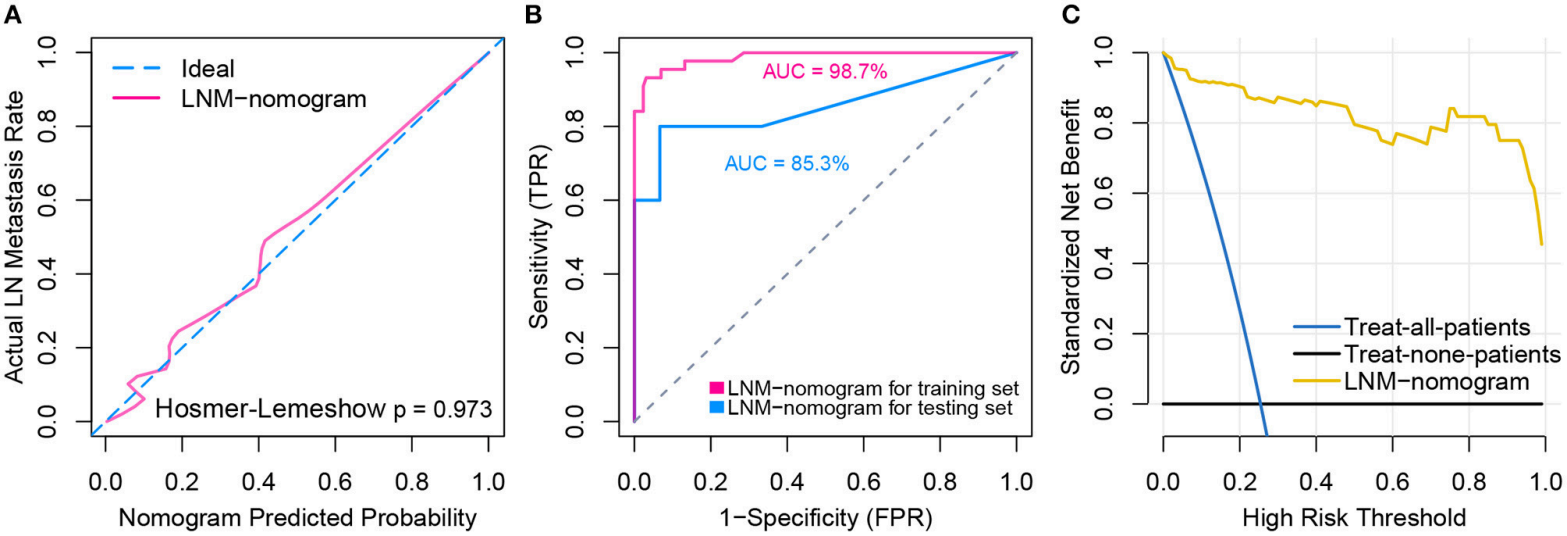
Model performance and clinical usefulness of the LNM-nomogram. **(A)** Calibration curve with Hosmer-Lemeshow test of the LNM-nomogram in the training set of TCGA-cohort. Calibration curve depicts the calibration of the fitted model in terms of the agreement between the predicted risk of LN metastasis and real observed outcomes. The x-axis represents the predicted LN metastasis risk and y-axis represents the actual LN metastasis rate. The pink solid line represents the performance of the LNM-nomogram, of which a closer fit to the diagonal dotted blue line represents an ideal prediction. The calibration curve was drawn by plotting $\hat{P}$ on the x-axis and $P_{c}={\left[1+exp\left(-\gamma_{0}+\gamma_{1}L\right)\right]}^{-1}$on the y-axis, where *P*_*c*_ is the actual probability, $L=logit\left(\hat{P}\right)$, $\hat{P}$ is predicted probability, γ_0_ is corrected intercept, and γ_1_ is slope estimates. **(B)** ROCs are created by plotting the true positive rate (TPR) against the false positive rate (FPR) at various threshold settings with corresponding AUCs labeled around the curves. **(C)** Decision curve analysis for the LNM-nomogram. The y-axis measures the net benefit. The yellow line represents the LNM-nomogram, the blue line represents the assumption that all patients have LN metastases and the black line on the bottom represents the assumption that no patients have LN metastases. The net benefit was calculated by subtracting the proportion of all patients who are false positive from the proportion who are true positive, weighting by the relative harm of forgoing treatment compared with the negative consequences of an unnecessary treatment. The relative harm was computed by *P*_*t*_/(1−*P*_*t*_). The threshold probability *P*_*t*_ is where the expected benefit of treatment is equal to the expected benefit of avoiding treatment; at which time a patient will opt for treatment informs us of how a patient weighs the relative harms of false positive results and false negative results ([a–c]/[b–d] = [1–*P*_*t*_]/*P*_*t*_) where [a–c] is the harm from a false negative result and [b–d] is the harm from a false positive result. Parameters of a, b, c, and d give the value of true positive, false positive, false negative, and true negative, respectively. The decision curve indicated that even if the threshold probability of a patient or doctor is really small, using the LNM-nomogram in the present study to predict LN metastases brings more benefit than treating either all or no patients.

Total points calculated by LNM nomogram for each sample in the testing set was determined to be a significant predictor when performing logistic regression (*p* = 0.032), and no departure from perfect fit was identified (*p* = 0.485) (Figure 6A, see Supplementary Figures S1A–T for total point of each testing sample). Internal validation obtained AUCs of 98.7 and 85.3% when deploying the LNM nomogram to the training set and the testing set (Figure 6B). The decision curve analysis showed that the LNM nomogram offered a net benefit over the “treat-all” or “treat-none” strategies at a really small threshold probability of a patient or doctor, which indicated that the LNM nomogram was clinically useful. For example, if the personal threshold probability of a patient is 60% (i.e., the patient would opt for treatment if his probability of LNM was >60%), then using the LNM nomogram to predict LN metastases could provide an added net benefit of 0.7386 compared to the “treat-all” or “treat-none” strategies (Figure 6C).

## Discussion

Bladder cancer ranks fourth in men and eighth in women among the most common malignancies in terms of frequency (27). However, little progress was made in the past decades toward prolonged survival of high-grade bladder cancer, leaving it still a lethal disease (28). Since a considerable amount of research has recognized LN involvement as the strongest independent prognostic variable for patient outcomes, proper identification of LN metastasis is of paramount importance (29). To date, several histopathologic findings that are known to be predictors of LN metastasis are commonly available post-operatively. Medical imaging has made great strides in pre-operative diagnosis, but the results are not fully satisfactory due to substantial false positives. Therefore, we sought here to develop and validate a diagnostic, LNM signature-based nomogram for pre-operative individualized prediction of LN metastasis in patients with bladder cancer. The nomogram offers an easy-to-use tool for pre-operative individualized prediction of LN metastasis and incorporates only two pre-operative items, LNM signature which stratifies patients by their risk of LN metastasis, and mutation status of *MLL2*. For the construction of the LNM signature, 180 candidate genes were reduced to 48 potential predictors by examining the predictor–outcome association by shrinking the regression coefficients with the LASSO algorithm. Compared to predictor selection based on strength of univariable association between predictor and outcome, this method further enables the combination of all selected features and creates a single signature, i.e., marker panels. Marker panels have been embraced in recent studies for multi-marker analysis (30, 31), such as a novel 6-microRNA-based model that was proposed to improve prognosis prediction of breast cancer (32), and a 6-DNA methylation signature that was recognized as a novel prognostic biomarker in ovarian serous cystadenocarcinoma (32). Moreover, the Oncotype DX is a 21-gene assay that represents the first clinically validated multi-gene assay which can quantify the likelihood of breast cancer recurrence (33, 34). Another 70-gene assay, MammaPrint, was developed by the Netherlands Cancer Institute and was used to predict the risk of developing metastasis within 5 years for breast cancer (35). Similarly, the LNM signature that combined multiple genes demonstrated adequate discrimination in the training set of TCGA cohort (AUC = 98.7%) and was satisfactory in the testing set (AUC = 85.3%). LNM signature was also presented as an independent predictor for overall survival in the TCGA cohort. In addition, supervised clustering using LNM signature enabled us to distinguish LN metastasis status in an independent GEO cohort (*p* = 0.048) and associated with patients' outcomes to some extent (*p* < 0.1). Thus, the non-invasive LNM signature allows for more convenient prediction of LN metastasis.

Note that mutation of *MLL2*, which was differentially mutated between LN+ and LN– tumors (7:46, *p* = 0.0166), was also a significant variable in the predictive model (*p* = 0.012). *MLL2*, also known as *KMT2D* (Lysine Methyltransferase 2D), of which mutation is a driver in numerous different cancer types resulting in transcription stress and genome instability (36), and a recent study demonstrated that MLL2 could sustain prostate carcinogenesis and metastasis (37). Because the LNM signature and the mutation of *MLL2* are available pre-operatively, our nomogram which generates individual probability by integrating the two factors provides clinicians and patients with a pre-operative individualized prediction of the LN metastasis risk, which is in line with the current trend toward personalized medicine (38).

Finally, and most importantly, the nomogram was designed to interpret individualized patient need for additional treatment or care. While the clinical consequences of a particular level of discrimination or degree of miscalibration could hardly be captured by risk prediction, discrimination, or calibration, a decision curve analysis assessing whether nomogram-assisted decision making improves patient outcomes was performed to justify the clinical usefulness of the LNM nomogram. This novel method offers insights into clinical consequences on the basis of threshold probability by deriving the net benefit (defined as the proportion of true positives minus the proportion of false positives weighted by relative harm of false-positive and false-negative results) (38, 39). Results showed that decisions based on the LNM nomogram yielded more favorable clinical consequences than the treat-all-patient scheme and the treat-none scheme, even given an extremely small threshold probability. However, our study harbored limitations, including the fact that radiomics characteristics and other pre-operative clinical features (e.g., hematuresis or not) were not considered under the existing framework. There has been tremendous growth in radiomics research in the past few years for assisting clinical diagnosis and improving predictive accuracy. An emerging field that is closely related to radiomics is radiogenomics, which integrates imaging and genomics data with the goal of improving patient stratification for precision medicine.

In short, this study presents a LNM nomogram incorporating a LNM signature and a genomic mutation, which can be conveniently used to facilitate pre-operative individualized prediction of LN metastasis in patients with bladder cancer.

## Data Availability

The datasets used and analyzed during the current study are available from the corresponding author on reasonable request.

## Author Contributions

XL and YW proposed the conception and design of this research. XL and LJ developed methodology. XL, BZ, and FY collected data and performed preprocessing. XL, WH, YZ, JW, XR, ZX, and XM analyzed and interpreted the data including statistical analysis, biostatistics, bioinformatics, and computational analysis. XL, JG, and YW were major contributors in writing the manuscript. All authors read and approved the final manuscript.

### Conflict of Interest Statement

The authors declare that the research was conducted in the absence of any commercial or financial relationships that could be construed as a potential conflict of interest.

## Abbreviations

LN: lymph node
LNM: lymph node metastasis
TPM: transcripts per kilobase million
FDR: false discovery rate
GSEA: gene set enrichment analysis
LASSO: Least absolute shrinkage and selection operator
OS: overall survival
PFS: progression-free survival
HR: hazard ratio
CI: confidence interval
ROC: operating characteristic curve
AUC: area under the curve.
