# Supplementary material for: A Pre-operative Nomogram for Prediction of Lymph Node Metastasis in Bladder Urothelial Carcinoma
Source: Front Oncol. 2019 Jun 21;9:488. doi: 10.3389/fonc.2019.00488 (PMC6598397; doi:10.3389/fonc.2019.00488)
Supplement: Supplementary file 1 [file Data_Sheet_1.pdf]

Supplementary Figure S1:

The total points of 20 testing samples calculated by LNM-nomogram and marked as red point, shown in sub-figure a) to t), respectively. Corresponding probability and its 95%CI of LN metastasis for each sample is pointing to the coordinate axis on the bottom with red arrow.

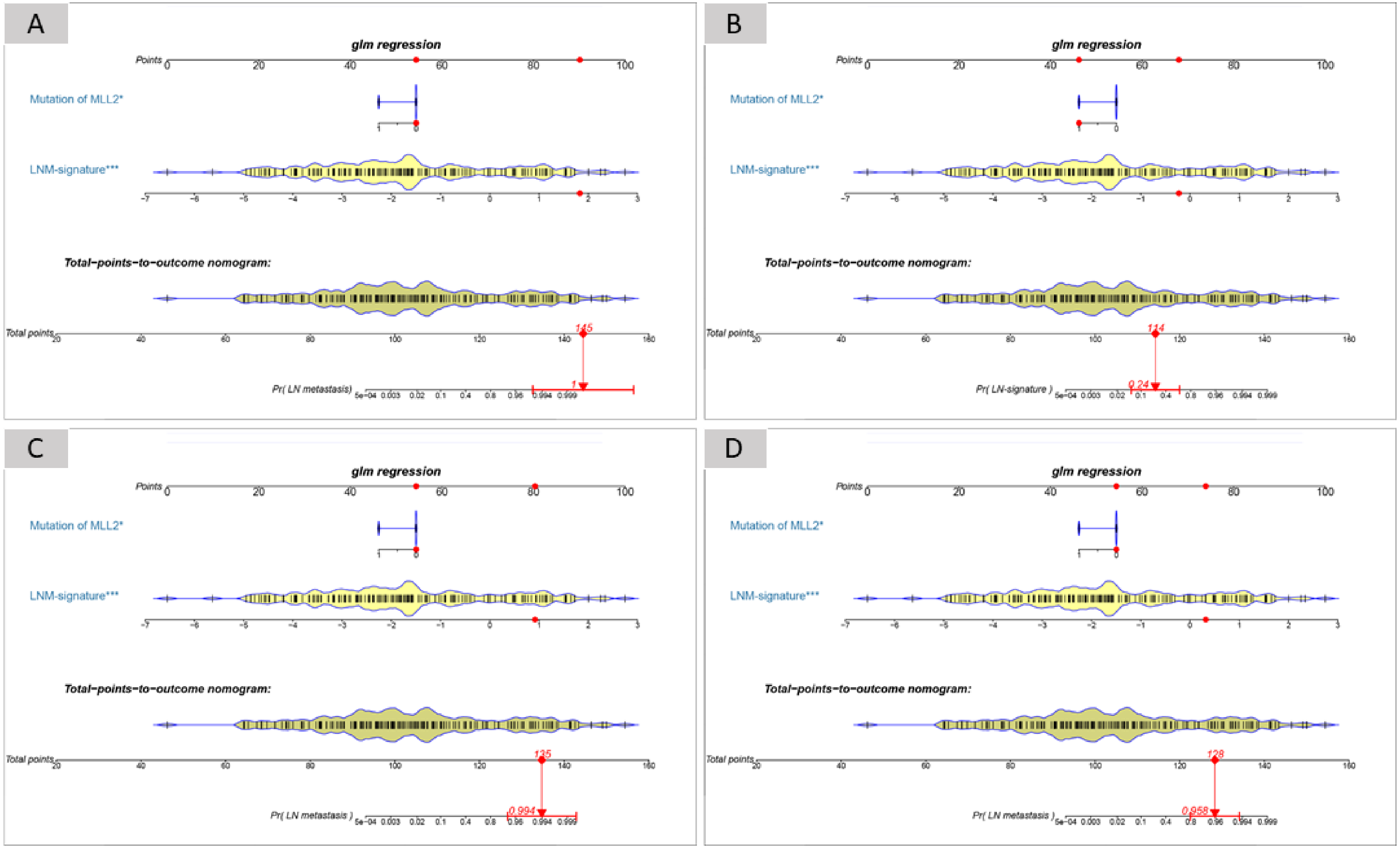
A
glm regression
Points
0
20
40
60
80
100
Mutation of MLL2*
LNM-signature***
Total−points−to−outcome nomogram:
Total points
145
1
Pr(LN metastasis)
B
glm regression
Points
Mutation of MLL2*
LNM-signature***
Total−points−to−outcome nomogram:
Total points
114
0.24
Pr(LN-signature)
C
glm regression
Points
Mutation of MLL2*
LNM-signature***
Total−points−to−outcome nomogram:
Total points
135
0.994
Pr(LN metastasis)
D
glm regression
Points
Mutation of MLL2*
LNM-signature***
Total−points−to−outcome nomogram:
Total points
128
0.958
Pr(LN metastasis)

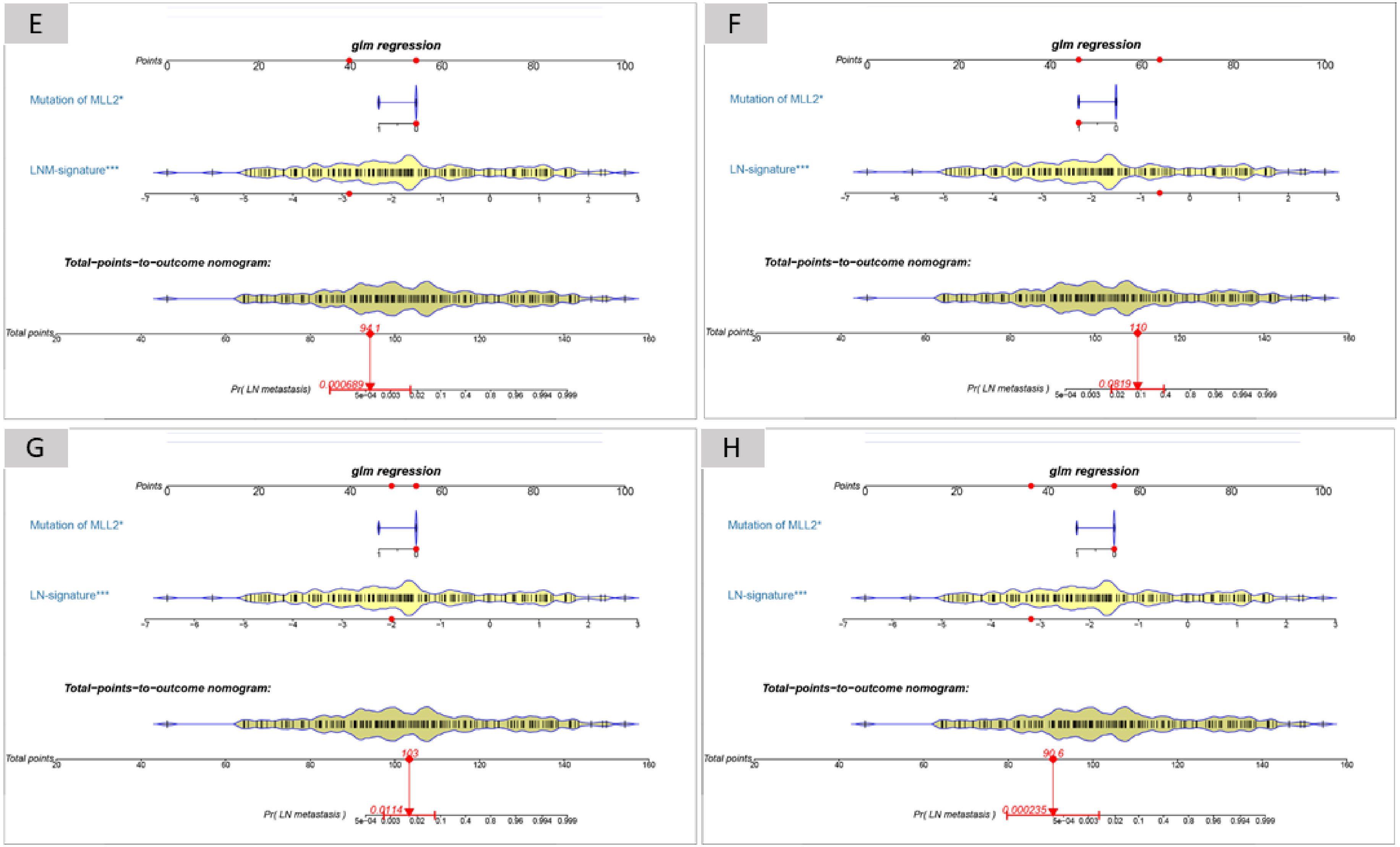
E
glm regression
Points
Mutation of MLL2*
LNM-signature***
Total-points-to-outcome nomogram:
Total points
94.1
Pr(LN metastasis)
0.000689
F
glm regression
Points
Mutation of MLL2*
LN-signature***
Total-points-to-outcome nomogram:
Total points
110
Pr(LN metastasis)
0.0819
G
glm regression
Points
Mutation of MLL2*
LN-signature***
Total-points-to-outcome nomogram:
Total points
103
Pr(LN metastasis)
0.0114
H
glm regression
Points
Mutation of MLL2*
LN-signature***
Total-points-to-outcome nomogram:
Total points
90.6
Pr(LN metastasis)
0.000235

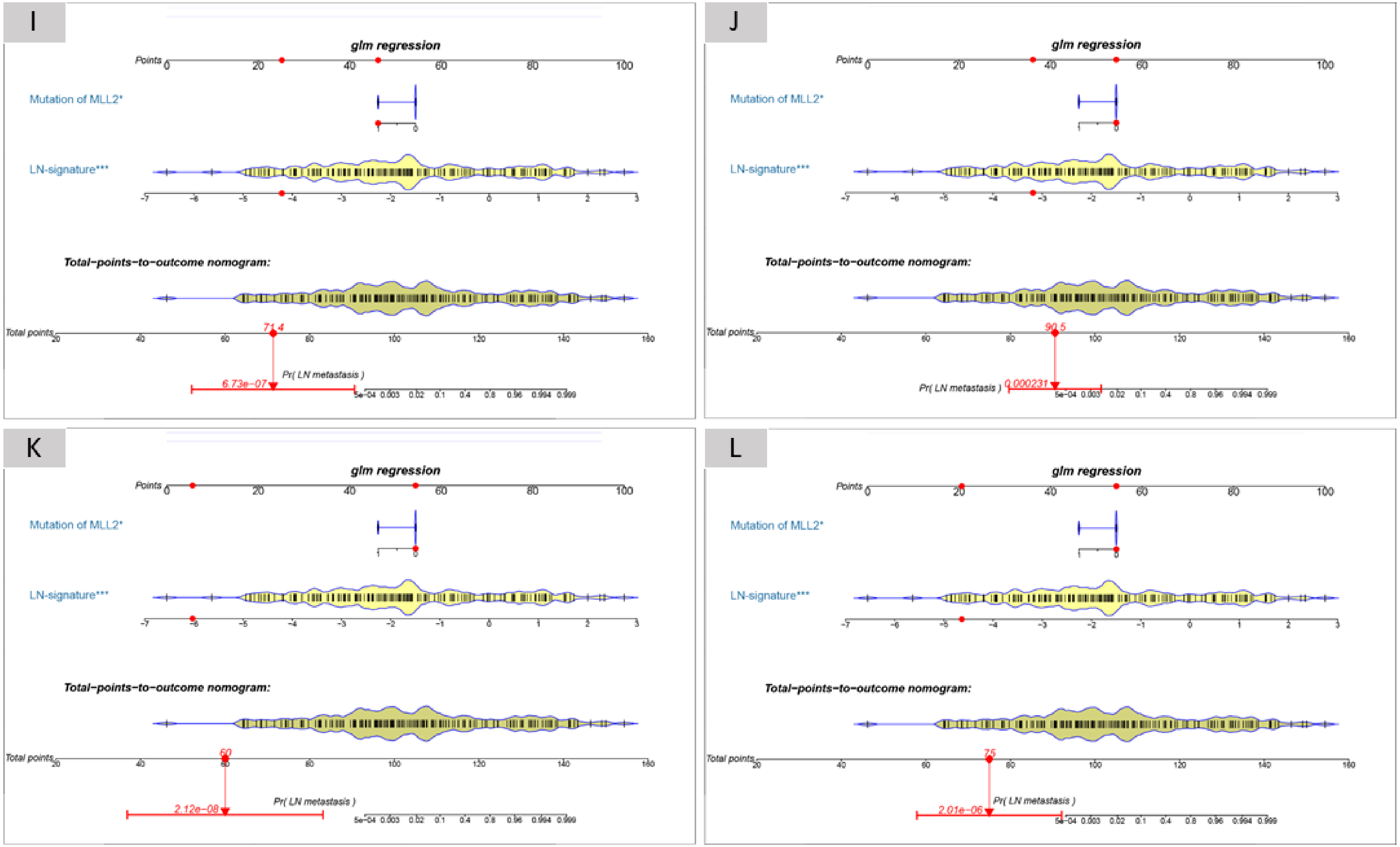
I
glm regression
Points
Mutation of MLL2*
LN-signature***
Total-points-to-outcome nomogram:
Total points
71.4
Pr(LN metastasis)
6.73e-07
J
glm regression
Points
Mutation of MLL2*
LN-signature***
Total-points-to-outcome nomogram:
Total points
90.5
Pr(LN metastasis)
0.000231
K
glm regression
Points
Mutation of MLL2*
LN-signature***
Total-points-to-outcome nomogram:
Total points
60
Pr(LN metastasis)
2.12e-08
L
glm regression
Points
Mutation of MLL2*
LN-signature***
Total-points-to-outcome nomogram:
Total points
75
Pr(LN metastasis)
2.01e-06

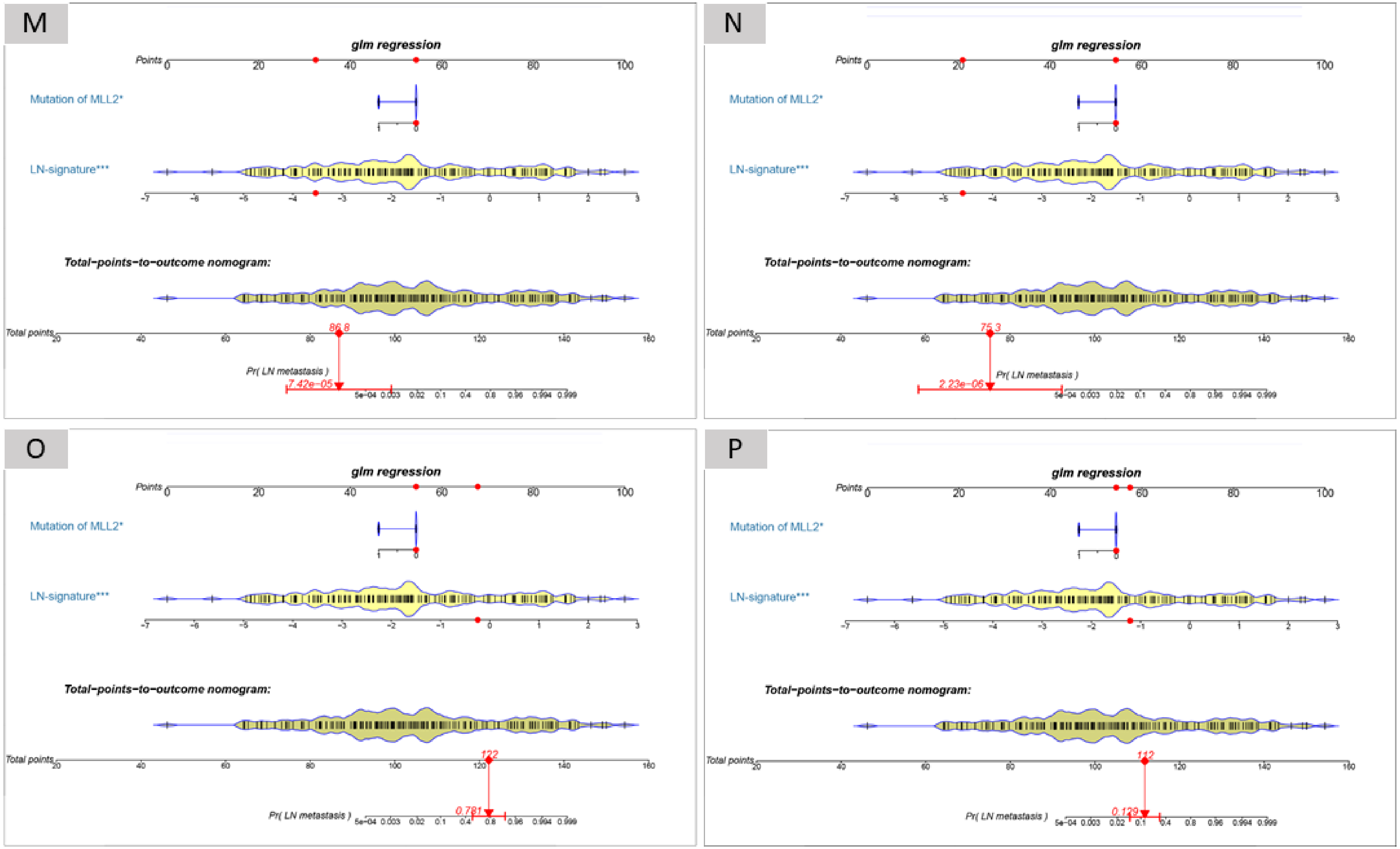
M
N
O
P
glm regression
Points
Mutation of MLL2*
LN-signature***
Total−points−to−outcome nomogram:
Total points
Pr(LN metastasis)
86.8
7.42e−05
75.3
2.23e−06
122
0.781
112
0.129

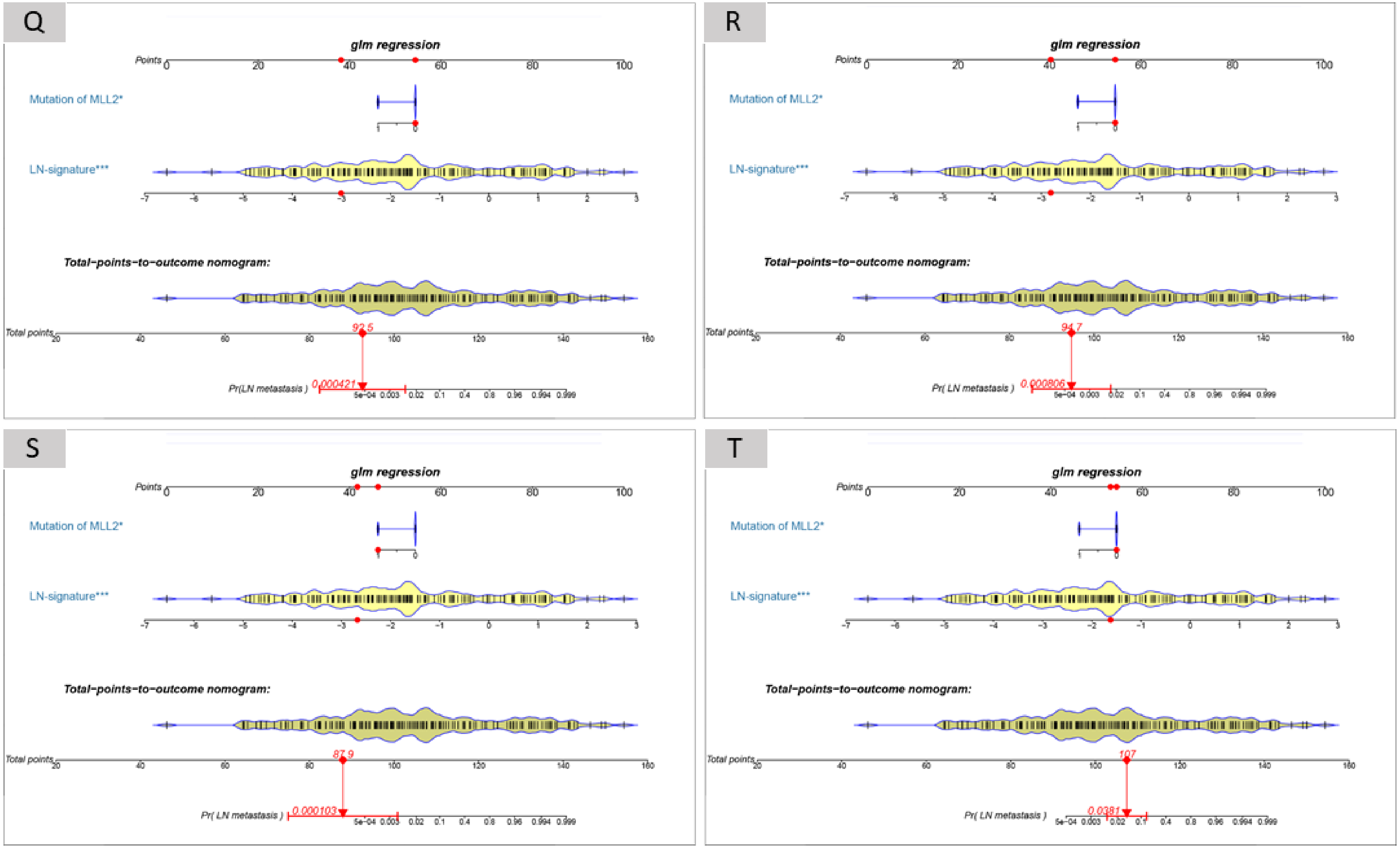

Q
glm regression
Points
Mutation of MLL2*
LN-signature***
Total−points−to−outcome nomogram:
Total points
92.5
Pr(LN metastasis)
0.000421
R
glm regression
Points
Mutation of MLL2*
LN-signature***
Total−points−to−outcome nomogram:
Total points
94.7
Pr(LN metastasis)
0.000806
S
glm regression
Points
Mutation of MLL2*
LN-signature***
Total−points−to−outcome nomogram:
Total points
87.9
Pr(LN metastasis)
0.000103
T
glm regression
Points
Mutation of MLL2*
LN-signature***
Total−points−to−outcome nomogram:
Total points
107
Pr(LN metastasis)
0.0381
